# A phase I study of AZD8186 in combination with docetaxel in patients with *PTEN*-mutated or *PIK3CB*-mutated advanced solid tumors

**DOI:** 10.1016/j.esmoop.2025.105569

**Published:** 2025-09-11

**Authors:** A.M. Schram, N. Takebe, A. Chen, Q. Zhou, A. Iasonos, J. Silber, M. Reynolds, S. Hussain, M. Gavriliuc, L.M. Smyth, D. Garrison, E.E. Dumbrava

**Affiliations:** 1 Memorial Sloan Kettering Cancer Center, New York, USA; 2 Weill Cornell Medical College, New York, USA; 3 National Cancer Institute, Bethesda, USA; 4 The University of Texas MD Anderson Cancer Center, Houston, USA; 5 Eli Lilly and Company, Indianapolis, USA; 6 Johns Hopkins Sidney Kimmel Comprehensive Cancer Center, Baltimore, USA

**Keywords:** solid tumor, docetaxel, AZD8186, neutropenia, PTEN, PIK3CB, basket trial

## Abstract

**Background:**

Loss of PTEN activity is common in solid tumors and promotes cancer growth through activation of the PI3K pathway. PTEN-deficient tumors have increased dependence on PI3Kβ and are sensitive to PI3Kβ inhibition in preclinical models. Efficacy is further enhanced by the addition of taxane chemotherapy. We conducted a phase I trial of AZD8186, a small molecule inhibitor of PI3Kβ and PI3Kδ, in combination with docetaxel (Taxotere) (NCI 10131; NCT03218826).

**Material and methods:**

Patients with advanced *PTEN*- or *PIK3CB*-mutated solid tumors identified through local testing were eligible. Treatment included docetaxel intravenously every 21 days and AZD8186 orally twice daily, 5 days on and 2 days off. Primary objectives were safety, tolerability, and maximum tolerated dose (MTD) as determined by a 3 + 3 dose-escalation design. Secondary objectives included assessment of antitumor activity.

**Results:**

Twenty-three patients were enrolled with 11 distinct tumor types across 5 dose levels. Clinically significant neutropenia led to dose-level adjustment and the addition of prophylactic growth factor. The MTD was not reached and AZD8186 120 mg twice daily with docetaxel 75 mg/m^2^ was named the recommended phase II dose. The most common treatment-emergent adverse events (TEAEs) were anemia (57%), diarrhea (43%), and fatigue (43%). The most common grade ≥3 TEAE was neutropenia (30%). One patient with docetaxel-naive prostate cancer had a prolonged partial response (overall response ratio 5.6%); clinical benefit rate was 22.2%.

**Conclusions:**

The combination of AZD8186 and docetaxel was generally well tolerated, with the exception of neutropenia, which was effectively managed with the use of growth factor. Limited clinical activity was observed.

## Highlights

• AZD8186 and docetaxel combination was well tolerated. A high rate of neutropenia was mitigated by prophylactic G-CSF.
• Future trials studying combination therapy with docetaxel should consider the inclusion of prophylactic G-CSF.
• *PTEN* deletion by next-generation sequencing strongly predicted for loss of PTEN protein by immunohistochemistry.
• Some patients may benefit from docetaxel + AZD8186, but there was no clear signal identified in this trial.

## Introduction

The PI3K/AKT/mTOR signaling network is a critical regulator of many cellular processes, including metabolism, proliferation, and survival.^1^ PI3K pathway dysregulation is a hallmark of various cancers and is seen in up to 50% of all solid tumors.^2^, ^3^, ^4^, ^5^, ^6^ Among other mechanisms, aberrant pathway activation can arise from somatic mutations in *PIK3CA* and *AKT1* or from loss/inactivation of the *PTEN* suppressor gene.^4^^,^^5^^,^^7^ *PTEN* loss is common in several tumor types including prostate, endometrial, and breast cancer.^4^^,^^5^

The PI3K lipid kinases are key regulators of the PI3K/AKT network and different Class IA isoforms (PI3Kα, PI3Kβ, and PI3Kδ with catalytic subunits p110α, p110β, and p110δ, respectively) play distinct roles. Furthermore, the PI3K enzymes are critically controlled by the tumor suppressor PTEN, a lipid phosphatase that reverses the action of PI3Ks.^8^ PTEN also has a nuclear role in promoting chromosome stability and DNA repair; therefore, loss of PTEN function increases genomic instability.^9^, ^10^, ^11^ Consequently, PTEN loss of function has been linked to poor outcome and therapeutic resistance in a number of cancers and represents a potential therapeutic target.^9^^,^^12^, ^13^, ^14^, ^15^, ^16^, ^17^, ^18^, ^19^, ^20^, ^21^, ^22^

PTEN-deficient tumors have increased dependence on PI3Kβ/AKT2 activity, requiring p110β for growth and signaling through the PI3K pathway and thus may be sensitive to PI3Kβ inhibition.^23^, ^24^, ^25^, ^26^ AZD8186 is a potent and selective small molecule inhibitor of PI3Kβ with additional activity against PI3Kδ. *In vitro*, AZD8186 inhibited PI3K signaling and viability in PTEN-null and PI3Kβ-driven preclinical models of breast and prostate cancer.^27^^,^^28^ The addition of paclitaxel chemotherapy resulted in synergistic apoptosis and growth inhibition.^27^ *In vivo*, AZD8186 had limited single-agent activity, which was significantly enhanced with the addition of paclitaxel or docetaxel in xenograft mouse models.^27^^,^^28^

Missense mutations in *PIK3CB*, the gene encoding the PI3Kβ protein, have been reported in cancer, where they can activate the PI3K/AKT pathway.^29^, ^30^, ^31^ Preclinical cancer models with the activating PIK3CB E1051K mutation are sensitive to PI3Kβ inhibition.^32^ We hypothesized that treatment with the PI3Kβ and PI3Kδ inhibitor AZD8186, in combination with docetaxel, represents a rational therapeutic strategy for patients with *PTEN*- or *PIK3CB*-mutated solid tumors. Docetaxel was chosen as the chemotherapeutic partner because diseases enriched for PTEN alterations such as prostate, endometrial, and breast cancer are commonly treated with docetaxel, in addition to the robust preclinical data in combination with AZD8186.

## Material and methods

### Study design and objectives

NCI 10131 (NCT03218826) was a multicenter, open-label, dose-finding clinical study of AZD8186 in combination with docetaxel (Taxotere; NSC# 628503) in patients with *PTEN*- or *PIK3CB*-mutated advanced solid tumors. Patients were recruited from four tertiary care centers through the Experimental Therapeutics Clinical Trials Network: Memorial Sloan Kettering Cancer Center, University of Texas MD Anderson Cancer Center, National Cancer Institute (NCI), and Johns Hopkins University Sidney Kimmel Comprehensive Care Center. The study used a 3 + 3 dose-escalation design. Baseline evaluations were conducted within 14 days before the start of therapy (30 days for radiographic assessment). Patients were treated in 21-day cycles and continued therapy until disease progression, unacceptable toxicity, death, or withdrawal of consent. An off-study evaluation was carried out 30 ± 7 days after the last dose.

The primary objectives of the phase I dose escalation were to assess the safety and tolerability, and to determine the maximum tolerated dose (MTD) and recommended phase II dose (RP2D) of AZD8186 and docetaxel. Secondary objectives included investigation of drug–drug interactions between AZD8186 and docetaxel, and assessment of antitumor activity measured by the overall response rate (ORR) and clinical benefit rate (CBR). ORR was defined as the rate of complete response (CR) or partial response (PR), and CBR was the proportion of patients with a CR, PR, and stable disease (SD) at 24 weeks as per RECIST v1.1.^33^ Patients with nonmeasurable disease were not assessable for CBR. Exploratory objectives included evaluation of PTEN protein expression and co-mutated genes in pretreatment tissue specimens and their association with treatment response or resistance, in addition to the description of emergent somatic mutations in cell-free DNA and postprogression tumor biopsies in patients who initially respond to and then progress on treatment.

The study was conducted in accordance with the principles of the Declaration of Helsinki, the International Council for Harmonization Good Clinical Practice guideline, and applicable regulatory requirements, including NCI Central Institutional Review Board approval. Of note, the study was initially designed to include three parts: dose escalation (part 1), pharmacodynamic dose expansion (part 2), and disease-specific dose expansion (part 3). After completion of part 1, parts 2 and 3 were not pursued due to discontinuation of AZD8186 development (NCI Protocol #10131, Version Dated 05/20/2021; Appendix A in the Supplementary Material, available at https://doi.org/10.1016/j.esmoop.2025.105569).

### Study population

Eligible patients were ≥18 years old with a histologically confirmed, unresectable or metastatic *PTEN*- or *PIK3CA*-mutated solid tumor for which standard curative or palliative measures do not exist. Qualifying *PTEN* alterations included loss of function and *PIK3CB* alterations included gain of function, as identified through local Clinical Laboratory Improvement Amendment (CLIA)-certified next-generation sequencing (NGS). Additional eligibility criteria included an Eastern Cooperative Oncology Group (ECOG) performance status of 0-2, adequate organ function, and adequate archival tissue or willingness to undergo a pretreatment biopsy.

Key exclusion criteria included HER2-positive breast cancer, prior treatment with PI3K or AKT inhibitors, known concurrent *RAF* or *PIK3CA* mutation, prior anticancer therapy within 4 weeks of treatment, known untreated or unstable brain metastases, ongoing use of strong CYP3A4 inhibitors and/or strong or moderate CYP3A4 inducers, and any condition associated with increased risk of bleeding. Patients with prior taxane use were eligible if they were anticipated to have maintained taxane sensitivity and in the opinion of the investigator would still benefit from docetaxel. The full eligibility is available in the supplementary files (Appendix A in the Supplementary Material, available at https://doi.org/10.1016/j.esmoop.2025.105569).

### Study treatment and dose escalation

Patients were treated with docetaxel intravenously on day 1 of each 21-day cycle. AZD8186 was administered orally twice daily (b.i.d.) in a 5 days on–2 days off schedule. The starting dose of AZD8186 60 mg b.i.d. was based on preliminary safety and pharmacodynamic data from the phase I monotherapy dose escalation of AZD8186 in patients with advanced solid tumors. AZD8186 was tolerable with dose-dependent target inhibition in plasma at 60 mg b.i.d. and 120 mg b.i.d.^34^ The starting dose of docetaxel was 75 mg/m^2^, chosen because this is a common standard dose used as a single agent and in combinations for patients with solid tumors. Prophylactic growth factor (GF) was initially prohibited; however, the study was later amended to require the use of GF per institutional guidelines during the first cycle of therapy and per the provider’s discretion on subsequent cycles. Dose modifications according to the study protocol were permitted in the event of a drug-related toxicity. A series of cohorts starting with three patients were planned per the standard 3 + 3 design (Appendix A in the Supplementary Material, available at https://doi.org/10.1016/j.esmoop.2025.105569). The MTD was defined as the highest dose level (DL) at which <33% of patients experienced a dose-limiting toxicity (DLT) during cycle 1 (the DLT-evaluable period).

### Safety

Patients were observed for the presence of DLTs during the first 21 days of treatment. A DLT was defined as any of the following (not attributable to the disease under investigation): grade 4 neutropenia lasting longer than 7 consecutive days despite GF support, grade 3 neutropenia with fever ≥38.5°C and/or systemic infection, grade 3 thrombocytopenia with bleeding, any other confirmed and clinically significant hematologic toxicity grade ≥4, nonhematologic laboratory abnormality grade ≥3, QTc prolongation grade ≥3, elevations of alanine aminotransferase or aspartate aminotransferase threefold or more above the upper limit of normal (ULN) with elevation of serum bilirubin greater than two times the ULN without evidence of cholestasis (alkaline phosphatase < ULN), and any event including significant dose reductions or omissions judged to be a DLT by the investigator. Patients who did not complete 75% of AZD8186 during the DLT period due to reasons other than drug toxicity were replaced. Safety assessments were conducted at baseline, weekly during the DLT evaluation period, and every 3 weeks thereafter.

### Efficacy assessment

Tumor response was evaluated locally based on RECIST v1.1 by computed tomography and/or magnetic resonance imaging, and bone scan (required only for patients with prostate cancer) carried out at screening and every 6 weeks for the first 24 weeks, then every 12 weeks thereafter.^33^ The best overall response was defined as the best response recorded from the start of treatment until disease progression. Objective response was considered confirmed if the response was maintained on a subsequent scan ≥4 weeks after the criteria for response were first met. Patients with prostate cancer were evaluated using a combination of RECIST v1.1 and the guidelines for prostate cancer endpoints developed by the Prostate Cancer Clinical Trials Working Group.^35^

### Immunohistochemistry

Tissue collected from pretreatment biopsy specimens or archival tissue underwent immunostaining for PTEN using the 138G6 Rabbit monoclonal antibody (#9559, Cell Signaling Technology; Danvers, MA). PTEN expression was quantified as absent if in ≤10% of tumor cells, intermediate if present in 11%-89%, and present if in ≥90%.

### Pharmacokinetics

Plasma samples were collected at predefined time points based upon the dosing schedules for docetaxel and AZD8186. Cycle 1, day 1 (C1D1) pretreatment baseline measurements were collected for both drugs. For docetaxel, samples were collected as follows: C2D1, pretreatment, 30 min and 55 min after the start of the docetaxel infusion, and 2 h and 5 h after the end of the docetaxel infusion; C2D2, ∼24 h after the start of the docetaxel infusion. For AZD8186, samples were collected as follows: C2D1, pretreatment and 1 h, 3 h, and 6 h after dosing; C2D2, ∼24 h after dose and before the next AZD8186 dose. Starting from cycle 3, a day 1 pretreatment sample was collected at each scheduled visit.

Plasma concentrations of docetaxel were quantified using a validated liquid chromatography and tandem mass spectrometry.^36^ The lower limit of quantitation for docetaxel was 0.5 ng/ml. AZD8186 plasma concentration was quantified using a validated ultraperformance liquid chromatography and tandem mass spectrometry method with a 5-2000 ng/ml dynamic range. Assays were carried out at the Sidney Kimmel Comprehensive Cancer Center Analytical Pharmacology Core Laboratory at Johns Hopkins University. Noncompartmental methods were used to describe steady-state predose concentration [concentration at the end of the dosing interval at steady state (Ctau,ss) AZD8186 only], maximum concentration (Cmax), time to maximum concentration (Tmax), area under the concentration–time curve from 0 h to 24 h (AUC 0-24 h) for docetaxel, and AUC 0-6 h for AZD8186, using Phoenix WinNonlin software (version 8.5, Certara, St. Louis, MO).

### Statistical methods

The sample size for dose escalation was determined by the 3 + 3 study design. The MTD and RP2D were descriptive. The frequency of adverse events is reported as percentages and described according to the NCI Common Terminology Criteria for Adverse Events Version 4. All patients who received any amount of study drug were assessable for toxicity. Efficacy analysis including ORR and CBR were descriptive and reported as a percentage with a two-sided exact 90% confidence interval (CI).

Statistical analysis for pharmacokinetic (PK) studies was carried out using Graph Pad Prism software version 9.4.1 (GraphPad Software, Inc., Boston, MA). PK parameters were evaluated statistically by Kruskal–Wallis test. Significance was determined at a level of *P* < 0.05. Between-group comparisons were conducted using the Wilcoxon signed rank test. Results pertaining to Cmax and AUC are presented as arithmetic mean values accompanied by standard deviation.

### Data availability

The data generated in this study are available upon request from the corresponding author.

## Results

### Patient characteristics and disposition

A total of 23 patients were enrolled between October 2018 and October 2021. The median age was 56 years (range 36-80 years) and 70% were female (Table 1). Most patients were white (91%) and had an ECOG performance score of 0 (39%) or 1 (57%). Patients had received a median of 4 prior lines of therapy (range 1-8) and 48% had received a prior taxane chemotherapy. The most common tumor types were prostate (22%), breast (17%), and colorectal cancer (13%). The majority of patients had alterations in *PTEN* (91%), including truncating nonsense or frameshift mutations (52%), homozygous deletions (17%), missense mutations (13%), and splice site mutations (9%). Three patients (13%) had alterations in *PIK3CB*, including two with a missense mutation and one with *PIK3CB* amplification. One patient with a *PIK3CB* missense mutation had two concurrent *PTEN*-truncating mutations.

**Table 1:** Patient demographic, disease, and genomic characteristics

Characteristic	Value
Median age, years (range)	56 (36-80)
Sex, *n* (%)	
Female	16 (70)
Male	7 (30)
Race, *n* (%)	
White	21 (91)
Black	1 (4)
Not reported	1 (4)
ECOG performance status, *n* (%)	
0	9 (39)
1	13 (57)
2	1 (4)
Primary tumor type, *n* (%)	
Prostate	5 (22)
Breast	4 (17)
Colorectal	3 (13)
Ovary	2 (9)
Uterine leiomyosarcoma	2 (9)
Endometrial	2 (9)
Neuroendocrine of vagina	1 (4)
Adenoid cystic carcinoma	1 (4)
Chondrosarcoma	1 (4)
Undifferentiated pleomorphic sarcoma	1 (4)
Stomach	1 (4)
Qualifying genomic alteration, *n* (%)	
*PTEN*	21 (91)
Truncating mutation	12 (52)
Deletion	4 (17)
Missense mutation	3 (13)
Splice site	2 (9)
*PIK3CB*	3 (13)
Missense mutation	2 (9)
Amplification	1 (4)
Multiple	1 (4)
Prior therapy	
Surgery	23 (100)
Chemotherapy	22 (96)
Radiation	13 (57)
Immunotherapy	6 (26)
Hormonal therapy	4 (17)
Vaccine therapy	1 (4)
Median prior lines of systemic therapy (range)	4 (1-8)

ECOG, Eastern Cooperative Oncology Group.

Patients were on the study for a median of 4 cycles (range 1-50). Reasons for discontinuation included radiographic disease progression (*n* = 14), clinical progression (*n* = 6), adverse events (*n* = 2), and withdrawal by the patient (*n* = 1).

### Dose escalation

**Figure 1:**
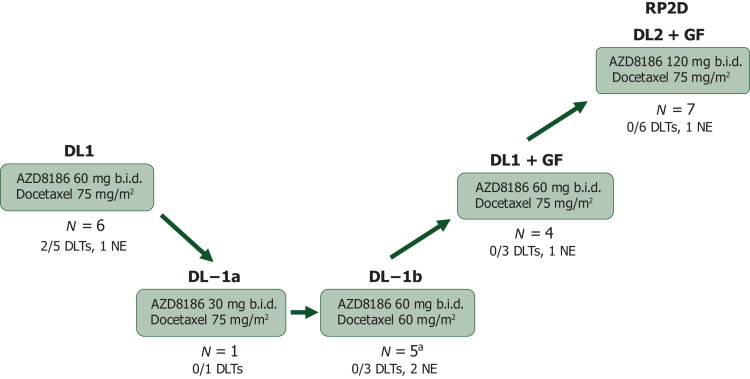
**The flowchart illustrates the dose escalation schema including patient accrual and dose-limiting toxicity (DLT) rate at each dose level.** DL, dose level; DLT, dose-limiting toxicity; GF, growth factor; NE, not evaluable; RP2D, recommended phase II dose. ^a^Prophylactic granulocyte-stimulating factor was added after four patients enrolled and the study was amended to allow dose re-escalation.

Five DLs were explored, differentiated by the dose of AZD8186, docetaxel, and/or the use of prophylactic GF (Figure 1; Supplementary Table S1, available at https://doi.org/10.1016/j.esmoop.2025.105569). DL 1 consisted of AZD8186 60 mg b.i.d. with docetaxel 75 mg/m^2^ every 3 weeks. Of six patients enrolled to DL1, two patients had DLTs including one patient with grade 3 edema and one patient with grade 3 febrile neutropenia. Per the protocol’s prespecified dose de-escalation rules, DL −1 was opened (AZD8186 30 mg b.i.d. with docetaxel 75 mg/m^2^ every 3 weeks) and enrolled one patient who did not experience a DLT. Given emerging data suggesting AZD8186 30 mg b.i.d. is subtherapeutic, a protocol amendment was issued opening DL −1b (AZD8186 60 mg b.i.d. with docetaxel 60 mg/m^2^ every 3 weeks). Four patients were initially treated at DL −1b. The frequency of neutropenia led to the implementation of mandatory prophylactic granulocyte colony-stimulating GF (G-CSF) injections going forward. In total, five patients were enrolled to DL −1b with no DLT in the three DLT-assessable patients. Improved tolerability with the use of prophylactic G-CSF allowed re-escalation to DL1 + GF. Four patients were enrolled and none of the three DLT-assessable patients experienced a DLT. The final dose explored, DL2 + GF, consisted of AZD8186 120 mg b.i.d. with docetaxel 75 mg/m^2^ every 3 weeks. Seven patients were enrolled and none of the six DLT-assessable patients experienced a DLT. The MTD was not reached and DL2 + GF was declared the RP2D.

### Safety

All patients developed at least one treatment-emergent adverse event (TEAE) (Table 2). The majority of nonlaboratory TEAEs were low grade (grade 1 or 2). The most common TEAEs across all DLs were anemia (57%), diarrhea (43%), fatigue (43%), anorexia (39%), nausea (39%), neutropenia (39%), and leukopenia (39%). Twenty patients (87%) experienced a treatment-emergent grade ≥3 toxicity. The most common grade ≥3 TEAEs were hematologic, including neutrophil count decreased (30%), decreased lymphocyte count (26%), decreased white blood cell count (26%), and anemia (13%). The use of prophylactic G-CSF decreased the rate of clinically significant neutropenia. Notably, one patient in the DL1 + GF cohort misunderstood directions and did not take G-CSF. Of the 12 patients who did not use prophylactic G-CSF, 6 developed grade ≥3 neutropenia (50%) compared with 1 of 11 patients (9%) treated using prophylactic G-CSF. Four patients (17%) required dose reduction of AZD8186 and five patients (22%) required reduction of docetaxel.

**Table 2:** Treatment-emergent adverse events observed in ≥15% of patients

Dose level	−1B (*N* = 5) All (%)	−1B (*N* = 5) ≥G3 (%)	−1 (*N* = 1) All (%)	−1 (*N* = 1) ≥G3 (%)	1 (*N* = 6) All (%)	1 (*N* = 6) ≥G3 (%)	1GF (*N* = 4) All (%)	1GF (*N* = 4) ≥G3 (%)	2 (*N* = 7) All (%)	2 (*N* = 7) ≥G3 (%)	Total (*N* = 23) All (%)	Total (*N* = 23) ≥G3 (%)
**Combined**	5 (100)	5 (100)	1 (100)	1 (100)	6 (100)	5 (83)	4 (100)	3 (75)	7 (100)	6 (86)	23 (100)	20 (87)
Anemia	4 (80)	0 (0)	1 (100)	0 (0)	2 (33)	1 (17)	2 (50)	0 (0)	4 (57)	2 (29)	13 (57)	3 (13)
Diarrhea	2 (40)	0 (0)	—	—	1 (17)	0 (0)	1 (25)	0 (0)	6 (86)	2 (29)	10 (43)	2 (9)
Fatigue	1 (20)	0 (0)	—	—	5 (83)	0 (0)	2 (50)	0 (0)	2 (29)	0 (0)	10 (43)	0 (0)
Anorexia	2 (40)	0 (0)	1 (100)	0 (0)	2 (33)	0 (0)	1 (25)	0 (0)	3 (43)	0 (0)	9 (39)	0 (0)
Nausea	1 (20)	0 (0)	—	—	2 (33)	0 (0)	2 (50)	0 (0)	4 (57)	0 (0)	9 (39)	0 (0)
Neutrophil count decreased	4 (80)	3 (60)	1 (100)	1 (100)	2 (33)	1 (17)	1 (25)	1 (25)	1 (14)	1 (14)	9 (39)	7 (30)
White blood cell decreased	4 (80)	3 (60)	1 (100)	1 (100)	3 (50)	1 (17)	—	—	1 (14)	1 (14)	9 (39)	6 (26)
Alkaline phosphatase increased	2 (40)	0 (0)	—	—	3 (50)	0 (0)	2 (50)	0 (0)	1 (14)	1 (14)	8 (35)	1 (4)
Hyperglycemia	3 (60)	0 (0)	—	—	2 (33)	0 (0)	2 (50)	0 (0)	1 (14)	0 (0)	8 (35)	0 (0)
Lymphocyte count decreased	3 (60)	3 (60)	1 (100)	1 (100)	—	—	1 (25)	0 (0)	3 (43)	2 (29)	8 (35)	6 (26)
Alopecia	1 (20)	0 (0)	—	—	1 (17)	0 (0)	2 (50)	0 (0)	3 (43)	0 (0)	7 (30)	0 (0)
Hyponatremia	2 (40)	1 (20)	—	—	2 (33)	1 (17)	—	—	3 (43)	1 (14)	7 (30)	3 (13)
AST increased	2 (40)	0 (0)	—	—	2 (33)	0 (0)	—	—	1 (14)	1 (14)	5 (22)	1 (4)
Fever	1 (20)	0 (0)	—	—	2 (33)	0 (0)	—	—	2 (29)	0 (0)	5 (22)	0 (0)
Hypokalemia	1 (20)	0 (0)	1 (100)	1 (100)	1 (17)	0 (0)	—	—	2 (29)	1 (14)	5 (22)	2 (9)
Vomiting	1 (20)	0 (0)	—	—	2 (33)	0 (0)	1 (25)	0 (0)	1 (14)	0 (0)	5 (22)	0 (0)
Abdominal pain	—	—	—	—	2 (33)	1 (17)	1 (25)	0 (0)	1 (14)	0 (0)	4 (17)	1 (4)
ALT increased	1 (20)	0 (0)	—	—	—	—	2 (50)	0 (0)	1 (14)	1 (14)	4 (17)	1 (4)
Creatinine increased	1 (20)	1 (20)	—	—	1 (17)	0 (0)	1 (25)	0 (0)	1 (14)	0 (0)	4 (17)	1 (4)
Edema limbs	1 (20)	0 (0)	—	—	3 (50)	1 (17)	—	—	—	—	4 (17)	1 (4)
Hypoalbuminemia	1 (20)	0 (0)	1 (100)	0 (0)	—	—	—	—	2 (29)	0 (0)	4 (17)	0 (0)
Peripheral sensory neuropathy	1 (20)	0 (0)	—	—	1 (17)	0 (0)	—	—	2 (29)	0 (0)	4 (17)	0 (0)

ALT, alanine aminotransferase; AST, aspartate aminotransferase; G3, grade 3; GF, growth factor.

Serious adverse events (SAEs) regardless of causality were reported in 13 patients (57%) (Supplementary Table S2, available at https://doi.org/10.1016/j.esmoop.2025.105569). Patients in all five DLs experienced DLTs. Four patients in DL1 (67%), one patient in DL −1 (100%), four patients in DL −1b (80%), one in DL1 + GF (25%), and three patients in DL2 (43%) experienced at least one SAE. The most common SAEs were hyponatremia, seen in four patients (17%), fever, and back pain observed in three patients each (13%).

Treatment-related AEs were observed in the majority of patients (96%), most commonly decreased neutrophil count (39%), diarrhea (39%), fatigue (39%), anemia (35%), decreased white blood cell count (35%), alopecia (26%), and nausea (26%) (Supplementary Table S3, available at https://doi.org/10.1016/j.esmoop.2025.105569). Seventy percent of patients had a grade ≥3 treatment-related AE, predominantly hematologic toxicity including decreased neutrophil count (26%), decreased white blood cell count (22%), and decreased lymphocyte count (13%). There were no grade 5 adverse events related to treatment. Dose reductions occurred in five patients (22%). Four patients dose-reduced AZD8186 (17%), and five patients dose-reduced docetaxel (22%). Two patients (9%) discontinued treatment due to treatment-related toxicity.

### Exploratory endpoints

Nineteen patients had sufficient tissue for PTEN evaluation by immunohistochemistry (IHC). Of the 17 patients with genomic *PTEN* alterations by NGS, 16 (94%) were negative for PTEN expression by IHC and 1 (6%) had intermediate expression. Intermediate expression was observed in one of two patients with a *PTEN* missense mutation. All 13 patients with *PTEN* deletions or truncating mutations had negative expression by IHC. PTEN IHC was negative in the two patients with *PTEN* splice site mutations. In the two patients with *PIK3CB* alterations, one with a *PIK3CB* missense mutation had normal PTEN expression (positive) and one with *PIK3CB* amplification had intermediate PTEN expression.

Due to the lack of efficacy observed, additional preplanned exploratory analyses could not be carried out. Specifically, we could not evaluate the relationship between co-mutational gene patterns and treatment response or resistance, or describe emergent somatic mutations in cell-free DNA and postprogression tumor biopsies at the time of acquired treatment resistance.

### Pharmacokinetics

Eighteen patients contributed PK data, with PK parameters summarized in Supplementary Figures S1 and S2 and Supplementary Tables S4 and S5, available at https://doi.org/10.1016/j.esmoop.2025.105569. Comparing docetaxel Cmax and AUC across DLs of AZD8186 (and vice versa), there was no apparent evidence of significant drug–drug interactions between docetaxel and AZD8186. Dose-adjusted values of Cmax and AUC did not differ across dose cohorts, roughly indicating dose proportionality (data not shown). Steady-state trough concentrations of AZD8186 across dosing cohorts were not significantly different.

### Clinical activity

**Figure 2:**
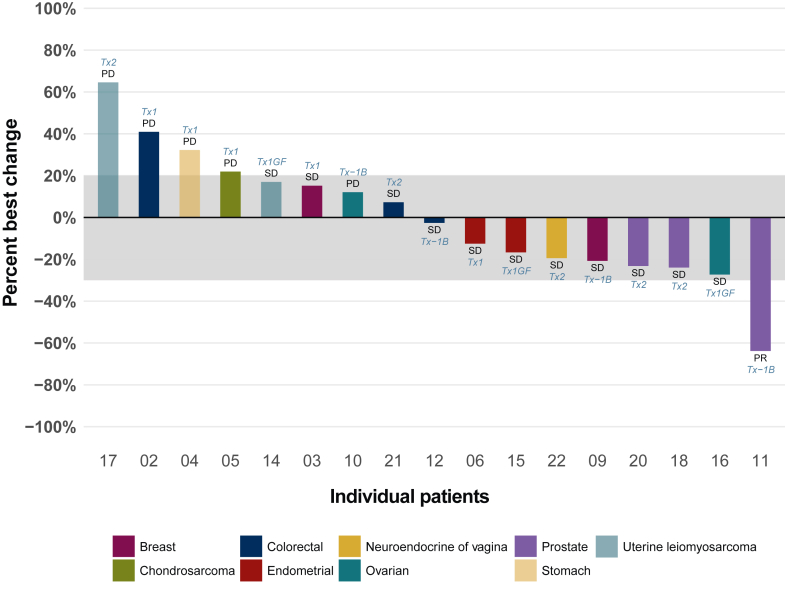
**Waterfall plot of best percentage change from baseline in tumor size per RECIST v1.1 criteria.** PD, progression of disease; PR, partial response; SD, stable disease; Tx, treatment.

**Figure 3:**
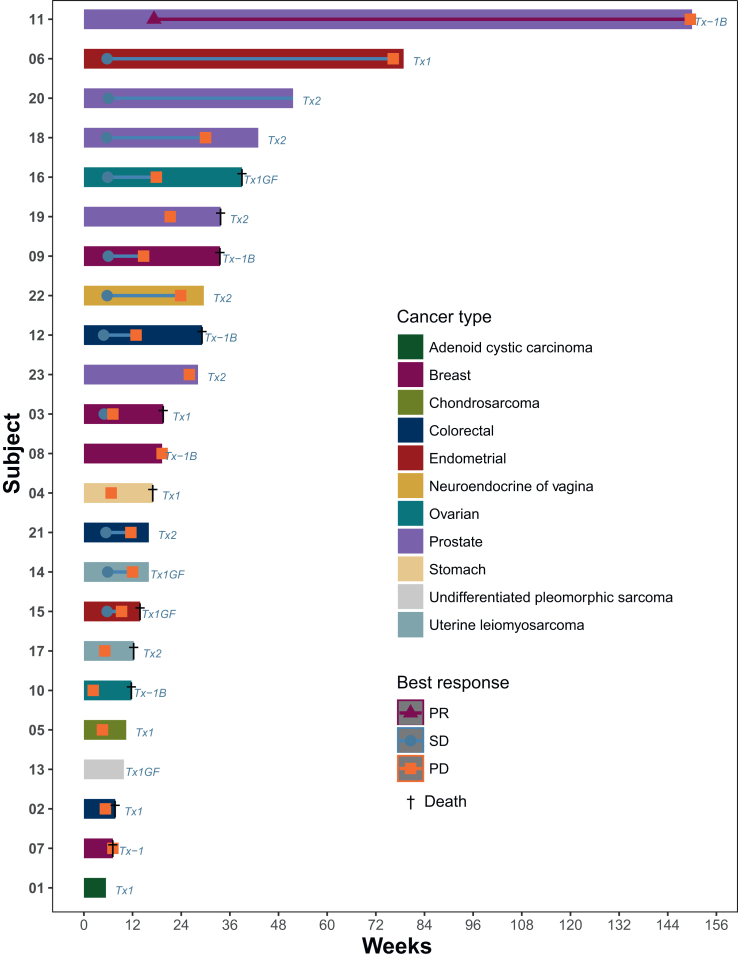
**Swimmer plot of patient outcomes and duration of response.** GF, growth factor; PD, progression of disease; PR, partial response; SD, stable disease; Tx, treatment.

Of the 18 efficacy-assessable patients, 1 patient had a PR (ORR 5.6%, 90% CI 0.3% to 23.8%) (Figure 2). The PR was in a patient in DL −1b with docetaxel-naive prostate cancer and a *PIK3CB* N717S mutation. The median progression-free survival (PFS) was 3 months (95% CI 1.6-4.9 months) (Figure 3). Clinical benefit was observed in 4 of 18 assessable patients (CBR 22.2%, 90% CI 8% to 43.9%). Among the total 23 patients, 19 progressed, 1 patient died before progression, and 3 patients were censored at 5.4 weeks, 9.9 weeks, and 51.6 weeks.

## Discussion

In this phase I study for patients with advanced *PTEN*- or *PIK3CB*-mutated solid tumors, we established the RP2D for the combination of AZD8186 (120 mg b.i.d., 5 days on and 2 days off) and docetaxel (75 mg/m^2^ every 3 weeks) with prophylactic G-CSF. The combination was generally well tolerated aside from a high rate of neutropenia. After the introduction of prophylactic G-CSF, the MTD was not reached.

Neutropenia is a well-characterized side-effect of docetaxel and the frequency observed here is in line with prior studies of docetaxel monotherapy.^37^, ^38^, ^39^ However, it is possible that AZD8186 also contributed to the risk of neutropenia. Although neutropenia was not frequently observed in the phase I study of AZD8186, P110δ is expressed on myeloid cells and neutropenia is common in clinical trials evaluating the PI3Kδ inhibitors idelalisib, copanlisib, and duvelisib.^34^^,^^40^, ^41^, ^42^ Future trials studying combination therapy with docetaxel should strongly consider the inclusion of prophylactic G-CSF.

We did not identify substantial docetaxel–AZD8186 PK interactions—a question of interest given that both drugs are CYP3A4 substrates. In addition, although the trough AZD8186 concentrations (Ctau,ss) did not vary across DLs, the 24-h postdose sample time and relatively short plasma half-life would predict trivial drug accumulation with repeat dosing and limited ability to detect significant differences in the troughs. These findings are limited by the very small sample size (1-7 patients) split among each of the six dose cohorts described in addition to a relatively small twofold or less dose range for either study drug and significant interindividual PK variability. Even so, the observed PK parameters for both docetaxel and AZD8186 were largely consistent with prior literature.^34^^,^^43^, ^44^, ^45^, ^46^

We demonstrate excellent concordance between genomic sequencing and protein expression. *PTEN* deletion or frameshift alterations by NGS strongly predicted for loss of PTEN protein by IHC. Evaluating the relationship between tumor sequencing and IHC has become increasingly important in clinical trials as the list of targetable biomarkers grows to include tumor suppressor loss and protein overexpression. Trials studying antibody–drug conjugates, for example, may enroll patients on the basis of NGS results, but concordance with IHC should be studied to validate the relationship between genomic alterations and target expression.

In this phase I study, the ORR was 5.6% (*n* = 1/18) and the CBR was 22.2% (*n* = 4/18). The one patient to respond had docetaxel-naive castration-resistant prostate cancer and a *PIK3CB* N717S variant of unknown significance. He remained on study without progression for nearly 3 years, a duration that far exceeds the expectation on docetaxel monotherapy.^47^ A patient with endometrial cancer harboring two PTEN loss-of-function alterations and the activating *PIK3CB* D1067Y mutation had tumor shrinkage and remained on study for >1 year without disease progression despite having previously been treated with taxane chemotherapy. Although these two cases are compelling, the antitumor activity was generally disappointing, and it is not possible to determine whether docetaxel monotherapy would have resulted in the limited efficacy observed. This study included a heavily pretreated and heterogeneous population with a diversity of disease types, genomic alterations, and DLs. It is possible that a subset of patients may benefit from the combination of docetaxel and AZD8186; there was, however, no clear signal identified in this trial. Similarly, a phase I clinical trial of the PI3Kβ inhibitor GSK2636771, and a subsequent phase I/II study of GSK2636771 with pembrolizumab, demonstrated only anecdotal responses in patients with castration-resistant prostate cancer with PTEN loss.^48^^,^^49^ A phase Ib/II study of weekly paclitaxel (days 1, 8, and 15 of a 28-day cycle) and AZD8186 in patients with *PTEN* or *PIK3CB*-altered gastric cancers had a 4-month PFS rate of 18.8% and was stopped due to futility.^50^

### Conclusions

The combination of AZD8186 and docetaxel was tolerable but demonstrated limited efficacy in patients with *PTEN*- or *PIK3CB*-mutated advanced solid tumors. Despite the clinical benefit observed in a subset of patients, the low response rate and discontinuation of the development of AZD8186 preclude further development of the regimen. Nonetheless, this study provides many important observations. We demonstrate the potential to study novel combinations of targeted therapy and chemotherapy in genomically selected patient populations, validate the use of NGS to select for patients with PTEN loss, and inform future trials of PI3Kβ inhibitors.
