# Supplementary material for: A phase I study of AZD8186 in combination with docetaxel in patients with PTEN-mutated or PIK3CB-mutated advanced solid tumors
Source: ESMO Open. 2025 Sep 11;10(9):105569. doi: 10.1016/j.esmoop.2025.105569 (PMC12529303; doi:10.1016/j.esmoop.2025.105569)
Supplement: Supplementary Material [file mmc1.docx]

**Supplementary Material**

**Supplementary Table 1.** Serious adverse events.

| **Dose Level** | DL 1  (N=6) | DL -1  (N=1) | DL -1B  (N=5) | DL1+ GF  (N=4) | DL 2  (N=7) | Total  (N=23) |
| --- | --- | --- | --- | --- | --- | --- |
| Combined | 4 (67%) | 1 (100%) | 4 (80%) | 1 (25%) | 3 (43%) | 13 (57) |
| Hyponatremia | 1 (17%) | - | 2 (40%) | - | 1 (14%) | 4 (17) |
| Fever | 2 (33%) | - | - | - | 1 (14%) | 3 (13) |
| Back pain | 1 (17%) | - | 2 (40%) | - | - | 3 (13) |
| Small intestinal obstruction | 1 (17%) | - | - | 1 (25%) | - | 2 (9) |
| Neutrophil count decreased | - | 1 (100%) | 1 (20%) | - | - | 2 (9) |
| Anorexia | - | 1 (100%) | 1 (20%) | - | - | 2 (9) |
| Febrile neutropenia | 1 (17%) | - | - | - | - | 1 (4) |
| Anemia | - | - | - | - | 1 (14%) | 1 (4) |
| Abdominal distension | 1 (17%) | - | - | - | - | 1 (4) |
| Abdominal pain | 1 (17%) | - | - | - | - | 1 (4) |
| Constipation | 1 (17%) | - | - | - | - | 1 (4) |
| Dysphagia | 1 (17%) | - | - | - | - | 1 (4) |
| Diarrhea | - | - | - | - | 1 (14%) | 1 (4) |
| Colitis | - | - | - | - | 1 (14%) | 1 (4) |
| Edema limbs | 1 (17%) | - | - | - | - | 1 (4) |
| White blood cell decreased | 1 (17%) | - | - | - | - | 1 (4) |
| Creatinine increased | - | - | 1 (20%) | - | - | 1 (4) |
| White blood cell decreased | - | - | 1 (20%) | - | - | 1 (4) |
| Hyperglycemia | 1 (17%) | - | - | - | - | 1 (4) |
| Dehydration | - | - | 1 (20%) | - | - | 1 (4) |
| Muscle weakness left-sided | - | 1 (100%) | - | - | - | 1 (4) |
| Pain in extremity | - | 1 (100%) | - | - | - | 1 (4) |

**Supplementary Table 2.** Treatment-related adverse events.

| **Dose Level** | **TAC-1B** | | **TAC-1** | | **TAC1** | | **TAC1+GF** | | **TAC2** | | **Total** | |
| --- | --- | --- | --- | --- | --- | --- | --- | --- | --- | --- | --- | --- |
|  | **(N=5)** | | **(N=1)** | | **(N=6)** | | **(N=4)** | | **(N=7)** | | **(N=23)** | |
| CTCAE v5.0 Term | **All (%)** | **≥G3 (%)** | **All (%)** | **≥G3 (%)** | **All (%)** | **≥G3 (%)** | **All (%)** | **≥G3 (%)** | **All (%)** | **≥G3 (%)** | **All (%)** | **≥G3 (%)** |
| *# Reporting AEs* | *4 (80)* | *4 (80)* | *1 (100)* | *1 (100)* | *6 (100)* | *4 (67)* | *4 (100)* | *2 (50)* | *7 (100)* | *5 (71)* | *22 (96)* | *16 (70)* |
| Diarrhea | 2 (40) | 0 (0) | - | - | - | - | 1 (25) | 0 (0) | 6 (86) | 1 (14) | 9 (39) | 1 (4) |
| Fatigue | 1 (20) | 0 (0) | - | - | 4 (67) | 0 (0) | 2 (50) | 0 (0) | 2 (29) | 0 (0) | 9 (39) | 0 (0) |
| Neutrophil count decreased | 4 (80) | 3 (60) | 1 (100) | 1 (100) | 2 (33) | 1 (17) | 1 (25) | 1 (25) | 1 (14) | 0 (0) | 9 (39) | 6 (26) |
| Anemia | 1 (20) | 0 (0) | - | - | 1 (17) | 0 (0) | 2 (50) | 0 (0) | 4 (57) | 2 (29) | 8 (35) | 2 (9) |
| White blood cell decreased | 4 (80) | 3 (60) | - | - | 3 (50) | 1 (17) | - | - | 1 (14) | 1 (14) | 8 (35) | 5 (22) |
| Alopecia | - | - | - | - | 1 (17) | 0 (0) | 2 (50) | 0 (0) | 3 (43) | 0 (0) | 6 (26) | 0 (0) |
| Nausea | - | - | - | - | 1 (17) | 0 (0) | 2 (50) | 0 (0) | 3 (43) | 0 (0) | 6 (26) | 0 (0) |
| Anorexia | 2 (40) | 0 (0) | - | - | 1 (17) | 0 (0) | 1 (25) | 0 (0) | 1 (14) | 0 (0) | 5 (22) | 0 (0) |
| Alanine aminotransferase increased | - | - | - | - | 1 (17) | 0 (0) | 2 (50) | 0 (0) | 1 (14) | 1 (14) | 4 (17) | 1 (4) |
| Alkaline phosphatase increased | 1 (20) | 0 (0) | - | - | - | - | 1 (25) | 0 (0) | 1 (14) | 1 (14) | 3 (13) | 1 (4) |
| Hyperglycemia | 2 (40) | 0 (0) | - | - | - | - | 1 (25) | 0 (0) | - | - | 3 (13) | 0 (0) |
| Hyponatremia | 1 (20) | 1 (20) | - | - | - | - | - | - | 2 (29) | 1 (14) | 3 (13) | 2 (9) |
| Lymphocyte count decreased | 2 (40) | 2 (40) | - | - | - | - | - | - | 1 (14) | 1 (14) | 3 (13) | 3 (13) |
| Mucositis oral | - | - | - | - | 2 (33) | 0 (0) | 1 (25) | 0 (0) | - | - | 3 (13) | 0 (0) |
| Peripheral sensory neuropathy | 1 (20) | 0 (0) | - | - | 1 (17) | 0 (0) | - | - | 1 (14) | 0 (0) | 3 (13) | 0 (0) |
| Abdominal pain | - | - | - | - | 1 (17) | 1 (17) | 1 (25) | 0 (0) | - | - | 2 (9) | 1 (4) |
| Aspartate aminotransferase increased | - | - | - | - | 1 (17) | 0 (0) | - | - | 1 (14) | 1 (14) | 2 (9) | 1 (4) |
| Dysgeusia | - | - | - | - | 1 (17) | 0 (0) | - | - | 1 (14) | 0 (0) | 2 (9) | 0 (0) |
| Edema limbs | - | - | - | - | 2 (33) | 1 (17) | - | - | - | - | 2 (9) | 1 (4) |
| Eye twitching | 1 (20) | 0 (0) | - | - | 1 (17) | 0 (0) | - | - | - | - | 2 (9) | 0 (0) |
| Vomiting | - | - | - | - | 1 (17) | 0 (0) | 1 (25) | 0 (0) | - | - | 2 (9) | 0 (0) |
| Weight loss | 1 (20) | 0 (0) | - | - | - | - | 1 (25) | 0 (0) | - | - | 2 (9) | 0 (0) |
| Ascites | - | - | - | - | 1 (17) | 0 (0) | - | - | - | - | 1 (4) | 0 (0) |
| Bloating | 1 (20) | 0 (0) | - | - | - | - | - | - | - | - | 1 (4) | 0 (0) |
| Blood bilirubin increased | 1 (20) | 0 (0) | - | - | - | - | - | - | - | - | 1 (4) | 0 (0) |
| Bone pain | - | - | - | - | 1 (17) | 0 (0) | - | - | - | - | 1 (4) | 0 (0) |
| Chills | - | - | - | - | - | - | - | - | 1 (14) | 0 (0) | 1 (4) | 0 (0) |
| Colitis | - | - | - | - | - | - | - | - | 1 (14) | 0 (0) | 1 (4) | 0 (0) |
| Constipation | - | - | - | - | 1 (17) | 0 (0) | - | - | - | - | 1 (4) | 0 (0) |
| Dehydration | 1 (20) | 1 (20) | - | - | - | - | - | - | - | - | 1 (4) | 1 (4) |
| Dyspepsia | - | - | - | - | - | - | - | - | 1 (14) | 0 (0) | 1 (4) | 0 (0) |
| Dyspnea | - | - | - | - | - | - | - | - | 1 (14) | 0 (0) | 1 (4) | 0 (0) |
| Esophagitis | - | - | - | - | - | - | - | - | 1 (14) | 0 (0) | 1 (4) | 0 (0) |
| Febrile neutropenia | - | - | - | - | 1 (17) | 1 (17) | - | - | - | - | 1 (4) | 1 (4) |
| Fever | - | - | - | - | - | - | - | - | 1 (14) | 0 (0) | 1 (4) | 0 (0) |
| Finger cramping | 1 (20) | 0 (0) | - | - | - | - | - | - | - | - | 1 (4) | 0 (0) |
| Gastritis | - | - | - | - | - | - | - | - | 1 (14) | 0 (0) | 1 (4) | 0 (0) |
| Hematuria | - | - | - | - | - | - | - | - | 1 (14) | 0 (0) | 1 (4) | 0 (0) |
| Hemoglobinuria | - | - | - | - | - | - | - | - | 1 (14) | 0 (0) | 1 (4) | 0 (0) |
| Hypertension | - | - | - | - | - | - | - | - | 1 (14) | 0 (0) | 1 (4) | 0 (0) |
| Hypoglycemia | 1 (20) | 0 (0) | - | - | - | - | - | - | - | - | 1 (4) | 0 (0) |
| Hypokalemia | - | - | - | - | - | - | - | - | 1 (14) | 0 (0) | 1 (4) | 0 (0) |
| Hypophosphatemia | - | - | - | - | - | - | - | - | 1 (14) | 0 (0) | 1 (4) | 0 (0) |
| Infusion related reaction | - | - | - | - | - | - | 1 (25) | 1 (25) | - | - | 1 (4) | 1 (4) |
| Localized edema | - | - | - | - | - | - | - | - | 1 (14) | 0 (0) | 1 (4) | 0 (0) |
| Muscle weakness lower limb | - | - | - | - | 1 (17) | 0 (0) | - | - | - | - | 1 (4) | 0 (0) |
| Myalgia | - | - | - | - | 1 (17) | 0 (0) | - | - | - | - | 1 (4) | 0 (0) |
| Nail discoloration | - | - | - | - | 1 (17) | 0 (0) | - | - | - | - | 1 (4) | 0 (0) |
| Nail Lifting | - | - | - | - | - | - | - | - | 1 (14) | 0 (0) | 1 (4) | 0 (0) |
| Nail loss | - | - | - | - | 1 (17) | 0 (0) | - | - | - | - | 1 (4) | 0 (0) |
| Oral pain | - | - | - | - | - | - | - | - | 1 (14) | 0 (0) | 1 (4) | 0 (0) |
| Platelet count decreased | - | - | - | - | - | - | - | - | 1 (14) | 0 (0) | 1 (4) | 0 (0) |
| Pleural effusion | - | - | - | - | - | - | - | - | 1 (14) | 0 (0) | 1 (4) | 0 (0) |
| Rash acneiform | - | - | - | - | 1 (17) | 0 (0) | - | - | - | - | 1 (4) | 0 (0) |
| Thromboembolic event | 1 (20) | 0 (0) | - | - | - | - | - | - | - | - | 1 (4) | 0 (0) |
| Thrush | 1 (20) | 0 (0) | - | - | - | - | - | - | - | - | 1 (4) | 0 (0) |
| Urinary retention | - | - | - | - | - | - | - | - | 1 (14) | 0 (0) | 1 (4) | 0 (0) |
| Weight gain | - | - | - | - | - | - | - | - | 1 (14) | 0 (0) | 1 (4) | 0 (0) |

**Supplementary Table 3.** Pharmacokinetic parameters of docetaxel.

| **Cohort** | **Docetaxel (mg/m^2^)** | **AZD8186 (mg)** | **C_max_ (ng/mL)** | **T_max_ (hour)** | **AUC_0-24h_ (ng*h/mL)** |
| --- | --- | --- | --- | --- | --- |
| DL -1B – docetaxel reduced | 45 | 30 | 1810 (1) | 0.92 (1) | 3496.7 (1) |
| DL -1B | 60 | 60 | 2060 (1) | 0.93 (1) | 3615.1 (1) |
| DL 1 – docetaxel reduced | 60 | 30, 60 | 2365.0 ± 190.9 (2) | 0.50 (2) | 4176.2 ± 562.7 (2) |
| DL 1 | 75 | 60 | 3536.7 ± 2485.3 (3) | 0.91 (0.45-1.27; 3) | 6981.7 ± 4457.9 (3) |
| DL 1+GF | 75 | 60 | 3483.3 ± 240.1 (3) | 0.74 (0.5-0.92; 3) | 4728.6 (1) |
| DL 2 | 75 | 120 | 2958.3 ± 186.9 (6) | 0.63 (0.47-0.92; 7) | 6493.9 ± 3437.8 (6) |

C_max_, maximum plasma concentration; T_max_: time to C_max_; AUC_0-24h_, area under the plasma concentration-time curve from 0 to 24 h.

Data are presented in the table as mean values ± SD (n). T_max_ is presented as median (range; n). If n < 3, the actual values are reported.

**Supplementary Table 4.** Pharmacokinetic parameters of AZD8186.

| **Cohort** | **Docetaxel** | **AZD8186** | **C_min,ss_** | **C_max_** | **T_max_** | **AUC_0-6h_** |
| --- | --- | --- | --- | --- | --- | --- |
|  | **(mg/m^2^)** | **(mg)** | **(ng/mL)** | **(ng/mL)** | **(hour)** | **(ng*h/mL)** |
| TAC-1B AZD8186 reduced | 45 | 30 | 130.0 (1) | 253.0 (1) | 8.43 (1) | 789.2 (1) |
| TAC-1B | 60 | 60 | 83.5 ± 55.1 (3) | 285.0 (1) | 4.75 (1) | 807.7 (1) |
| TAC-1 AZD8186 reduced | 60 | 30 | 66.0 (1) | NC | NC | NC |
| TAC-1 | 75 | 60 | 149.8 ± 77.2 (3) | 464 | 3.87 (1) | 1105.5 (1) |
| TAC1+GF | 75 | 60 | 176.0 (1) | 165.5 | 2.76 (3) | 224.2 (3) |
| TAC-2 | 75 | 120 | 76.8 ± 49.2 (6) | 531.8 | 4.43 (7) | 1796.1 (7) |

C_min,ss_, steady-state trough concentration; C_max_, maximum plasma concentration; T_max_: time to C_max_; AUC_0-6h_, area under the plasma concentration-time curve from 0 to 6 h; NC, not calculated.

Data are presented in the table as mean values ± SD (n). If n < 3, the actual values are reported.


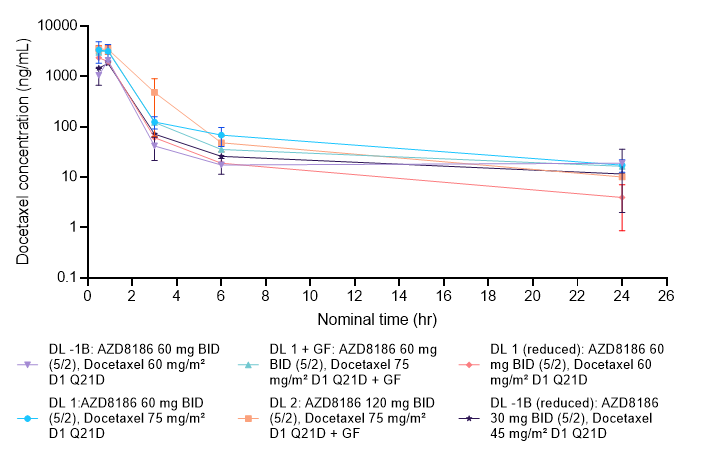


**Supplementary Figure 1.** Docetaxel concentration-time profile on C2D1 across different dose levels of AZD8186 and docetaxel. The graph depicts mean docetaxel plasma concentrations (ng/mL) over a 24-hour period following administration on Day 1 of a 21-day cycle. Concentrations are presented on a logarithmic scale, with time (hours) on the x-axis. Error bars represent the standard error of the mean (SEM).


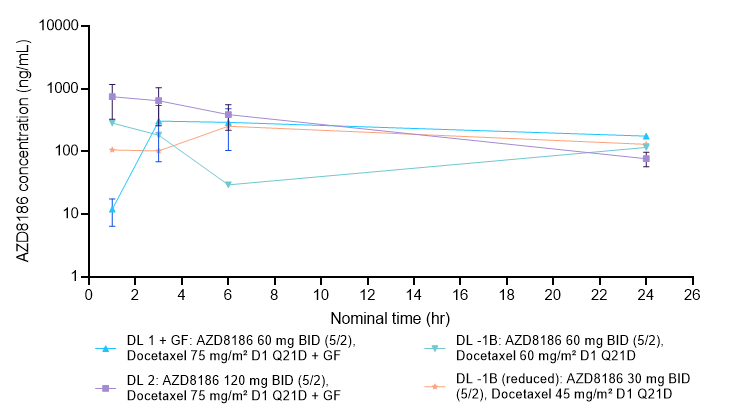


**Supplementary Figure 2**. AZD8186 concentration-time profile on C2D1 across different dose levels of AZD8186 and docetaxel. The graph displays mean AZD8186 plasma concentrations (ng/mL) over a 24-hour period, plotted on a logarithmic scale, with nominal time (hours) on the x-axis. Error bars represent the standard error of the mean (SEM).
